# A prospective development study investigating focal irreversible electroporation in men with localised prostate cancer: Nanoknife Electroporation Ablation Trial (NEAT)

**DOI:** 10.1016/j.cct.2014.07.006

**Published:** 2014-09

**Authors:** Massimo Valerio, Louise Dickinson, Afia Ali, Navin Ramachandran, Ian Donaldson, Alex Freeman, Hashim U. Ahmed, Mark Emberton

**Affiliations:** a Division of Surgery and Interventional Science, University College London, London, UK; b Department of Urology, University College London Hospitals NHS Foundation Trust, London, UK; c Department of Urology, Centre Hospitalier Universitaire Vaudois, Lausanne, Switzerland; d Department of Mental Health Sciences, University College London, London, UK; e Department of Radiology, University College London Hospitals NHS Foundation Trust, London, UK; f Department of Histopathology, University College London Hospitals NHS Foundation Trust, London, UK

**Keywords:** Focal therapy, Health technology assessment, Irreversible electroporation, Prostate cancer

## Abstract

**Introduction:**

Focal therapy may reduce the toxicity of current radical treatments while maintaining the oncological benefit. Irreversible electroporation (IRE) has been proposed to be tissue selective and so might have favourable characteristics compared to the currently used prostate ablative technologies. The aim of this trial is to determine the adverse events, genito-urinary side effects and early histological outcomes of focal IRE in men with localised prostate cancer.

**Methods:**

This is a single centre prospective development (stage 2a) study following the IDEAL recommendations for evaluating new surgical procedures. Twenty men who have MRI-visible disease localised in the anterior part of the prostate will be recruited. The sample size permits a precision estimate around key functional outcomes. Inclusion criteria include PSA ≤ 15 ng/ml, Gleason score ≤ 4 + 3, stage T2N0M0 and absence of clinically significant disease outside the treatment area. Treatment delivery will be changed in an adaptive iterative manner so as to allow optimisation of the IRE protocol. After focal IRE, men will be followed during 12 months using validated patient reported outcome measures (IPSS, IIEF-15, UCLA-EPIC, EQ-5D, FACT-P, MAX-PC). Early disease control will be evaluated by mpMRI and targeted transperineal biopsy of the treated area at 6 months.

**Discussion:**

The NEAT trial will assess the early functional and disease control outcome of focal IRE using an adaptive design. Our protocol can provide guidance for designing an adaptive trial to assess new surgical technologies in the challenging landscape of health technology assessment in prostate cancer treatment.

## 1 Introduction

Recent evidence from large randomised controlled trials (RCTs) in prostate cancer has challenged the current diagnostic and treatment pathway of the disease [1], [2]. This is due to an unfavourable benefit/risk ratio. This is because of two reasons. First, many men are diagnosed with indolent prostate cancer which do not impact on his quality of life or life expectancy [3], [4]. Second, when treatment is given, it is applied in a radical whole-gland manner (using surgery or radiotherapy) which causes collateral tissue damage and side effects. In summary, erectile dysfunction, urinary incontinence and bowel toxicity occur in about 40–95%, 10–20% and 5–35% of men undergoing radical therapy, respectively [5].

As a result, one strategy that has been proposed to mitigate the harms of the current pathway is focal therapy. This involves targeting therapy to the area of the prostate harbouring clinically significant disease (cancer that is not indolent and requires treatment), while sparing the rest of the gland. Indeed, by preserving prostatic tissue and the important structures surrounding the prostate — such as external urinary sphincter, neurovascular bundles, bladder neck, and rectum — the toxicity profile decreases significantly. A recent systematic review has shown that various sources of energy have been used for focal therapy [6]. Overall, erectile dysfunction, urinary incontinence and bowel toxicity were lower and ranged from 0 to 46%, 0 to 5% and 0 to 33%, respectively [6]. Cancer-control outcomes demonstrated residual cancer in 4–50% of men having a biopsy after treatment, although only 0–17% of residual disease was deemed clinically significant [6].

Currently, most of the energy sources used in a focal manner utilise a thermal effect to destroy prostatic tissue: cryotherapy uses temperatures below − 40 °C and high-intensity focused ultrasound therapy (HIFU) use temperatures above + 60 °C [6], [7]. Thermal tissue destruction may have some drawbacks. First, it is non-selective towards the different structures (nerves, stroma, vasculature, glands) of the prostate and collateral damage could still occur. Second, especially with high temperatures, the heat-sink effect of intra- and extra-prostatic vessels (which can dissipate the energy) can lead to under-treatment. Third, the precision required to treat an area of prostate to within millimetre accuracy may be lacking. New technologies in the field might combine better cancer control outcomes with enhanced tissue preservation.

Irreversible electroporation (IRE) is a promising new technology. By using low voltage direct electric current, IRE permanently damages the cell membrane, and leads to cell death with no thermal effect [8]. IRE has been used for the treatment of localised and metastatic tumours in other solid organ malignancies such as kidney, liver, pancreas and lung [9]. It has some potential advantages that might lend itself to focal therapy in prostate cancer. First, the tissue outside the electrical field is theoretically not compromised since there is no effect in those areas [8]. Second, the treatment has shown tissue-selectivity in pre-clinical studies, so that collagenous structures — such as vessels, nerves and the urethra — seem not to be affected [8], [10], [11].

As a result, we hypothesised that IRE would lead to low rate of side-effects when applied in a focal manner to men with clinically significant localised prostate cancer. To date, IRE has been used only in one proof of concept study with no intention to treat [12]. As a consequence, we followed the IDEAL guidelines for evaluating surgical innovation which recommends stages of evaluation and is mirrored upon the UK Medical Research Council's (MRC) Guidelines of evaluation complex interventions [13], [14].

The optimal trial design for ablative therapies has been debated and discussed in detail by numerous consensus groups of clinicians and methodologists [15], [16], [17]. The FDA in the US has also recently held a panel discussion in 2013 to look into this area [18]. The key problem has been in deciding on a trial design that shows benefit to patients. While side-effects could be measured in the short-term, disease control in a cancer which has a long natural history, is difficult to determine objectively. Our NEAT protocol, we believe, provides an exemplar of an adaptive design that would potentially answer the question about whether there is benefit in terms of reduced side-effects as well as provide robust data on the disease control outcomes in the short and medium term so that novel therapies can be approved in a timely manner to benefit men with prostate cancer.

## 2 NEAT protocol

### 2.1 Study management

NEAT is an investigator led single arm interventional adaptive trial compliant to the MRC guidelines for evaluating complex procedures, and to stage 2a (prospective development study) according to the IDEAL guidelines. Investigators from University College London (UCL) designed the protocol, considering feedback from the National Cancer Research Institute (UK) Prostate Clinical Studies Group as well as from patient representatives. The study is sponsored by UCL, and will be run at the University College London Hospital. The Joint Research Office of the local Research & Development unit is responsible for monitoring patients' safety and adherence to good clinical practice. The trial steering committee (TSC) is composed of an independent chair, the co-principal investigators, the study coordinators, a patient representative, two experts in the field and the study statistician. The independent data monitoring committee (IDMC) includes an independent chair expert in the field of prostate cancer therapy, a trial unit manager and a senior statistician (all of whom are independent of the study). This trial is registered in the clinicaltrials.gov database (NCT01726894).

### 2.2 Study population eligibility

Men with histologically proven MRI-visible prostate cancer localised in the anterior part of the prostate. Therefore, only men with disease in the transition area, the anterior fibromuscular stroma or the peripheral zone of the prostate located in front of the urethra will be considered eligible. Presence of clinically significant disease outside the treated area represents an exclusion criteria, but insignificant disease left untreated is acceptable. A complete list of the eligibility criteria is given in Table 1.

**Table 1:** Inclusion and exclusion criteria for the NEAT trial.

*Inclusion criteria*
• Histologically proven prostate cancer, Gleason score ≤ 7.
• An anterior visible lesion on mpMRI, that is accessible to Irreversible Electroporation.
• Transperineal prostate biopsies (template mapping and/or zonal and targeted) correlating with clinically significant lesion in the area of the MR-visible lesion.
• Absence of clinically significant histological disease outside of the planned treatment zone.
• Stage radiological T1-T3aN0M0 disease, as determined by local guidelines.
• Serum PSA ≤ 15 ng/ml.
• Age ≥ 40 years and life expectancy of ≥ 10 years.
• Signed informed consent by patient.
• An understanding of the English language sufficient to understand written and verbal information about the trial and consent process.

*Exclusion criteria*
• Men who have had previous radiation therapy to the pelvis.
• Men who have had androgen suppression/hormone treatment within the previous 12 months for their prostate cancer.
• Men with evidence of metastatic disease or nodal disease outside the prostate on bone scan or cross-sectional imaging.
• Men with a non-visible tumour on mpMRI.
• Men with an inability to tolerate a transrectal ultrasound.
• Men with latex allergies.
• Men who have undergone prior significant rectal surgery preventing insertion of the TRUS probe (decided on the type of surgery in individual cases).
• Men who have had previous NANOKNIFE, HIFU, cryosurgery, thermal or microwave therapy to the prostate.
• Men who have undergone a Transurethral Resection of the Prostate (TURP) for symptomatic lower urinary tract symptoms within the prior 6 months. These patients may be included within the trial if deferred from consent and screening until at least 6 months following the TURP.
• Men not fit for major surgery as assessed by a consultant anaesthetist.
• Men unable to have pelvic MRI scanning (severe claustrophobia, permanent cardiac pacemaker, metallic implant etc likely to contribute significant artefact to images).
• Presence of metal implants/stents in the urethra.
• Men with renal impairment with a GFR of < 35 ml/min (unable to tolerate Gadolinium dynamic contrast enhanced MRI).

In this study, we use a definition specifically derived for transperineal biopsies, as those definitions based on transrectal biopsies have no proven validity in biopsies taken transperineally. Our definition is based on the presence of any positive core with primary or secondary Gleason pattern ≥ 4 (i.e., 3 + 4 or 4 + 3), and/or a maximum cancer core length ≥ 4 mm. Insignificant disease is defined as opposite as Gleason 3 + 3 with maximum cancer core length of 3 mm or less.

### 2.3 Trial design

**Fig. 1:**
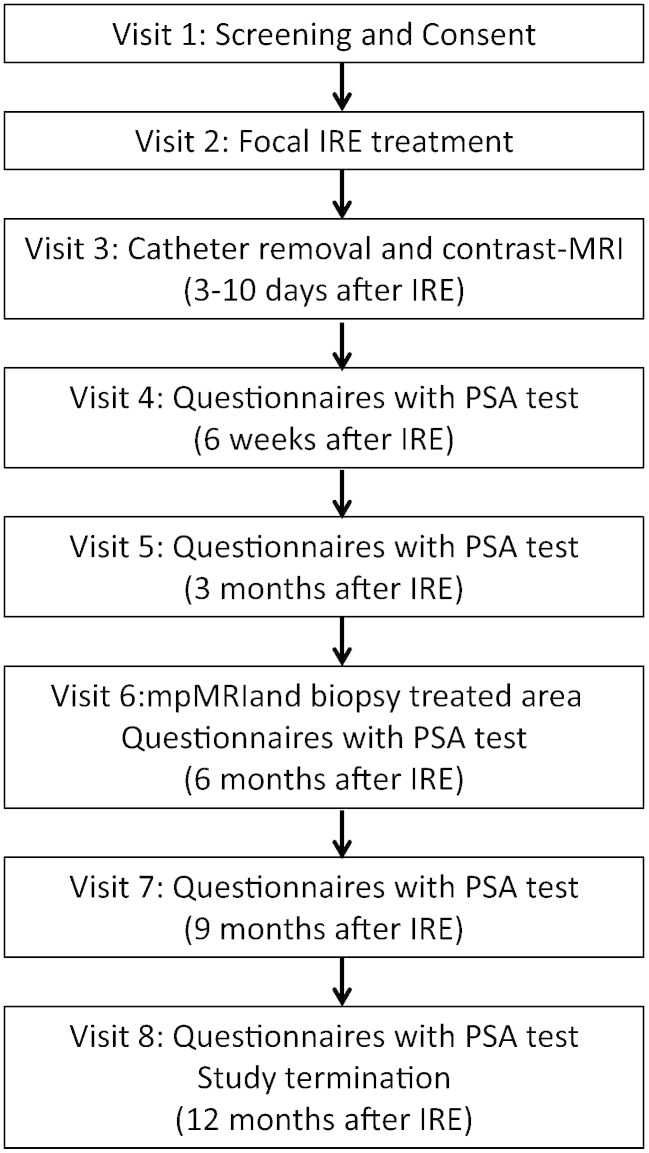
Trial flow.

**Fig. 2:**
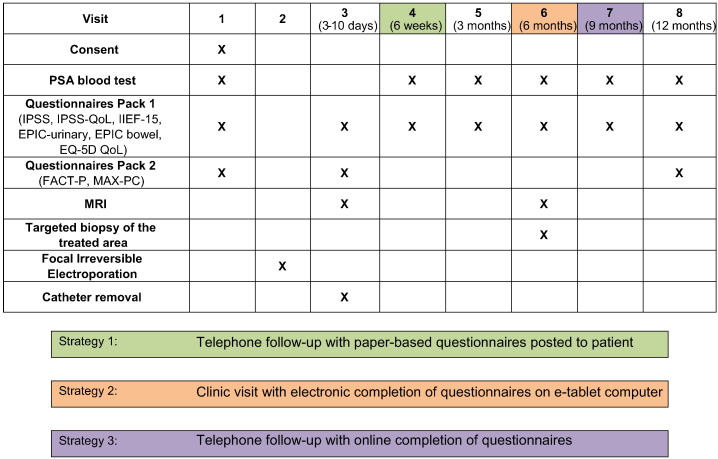
Single visit schedule throughout the trial. Patient in the embedded qualitative study will have visits 4, 6 and 7 carried out in an alternative electronic manner, as shown in the table and legends.

The trial flow and the visit schedule are displayed in Fig. 1, Fig. 2, respectively.

#### 2.3.1 Trial entry

It is of key importance to well select men who are likely to benefit from focal treatment in which significant disease is localised in only one part of the gland. To avoid the possibility of leaving clinically significant cancer untreated, all patients undergone state-of-the-art accurate imaging and tissue sampling. Only men in which imaging and histology are concordant in localising disease in the anterior part of the prostate were eligible.

A mpMRI will be performed in a 1.5 Tesla or 3 Tesla scanner and a pelvic phased array receiver with a pelvic coil using a standardized protocol as described elsewhere [19]. The protocol includes T2-weighted, dynamic contrast enhanced and diffusion-weighted images. The sequences used and the reporting method will follow those laid down by the European society of uro-radiology [20]. The images will be evaluated and reported by experienced radiologists who will score the likelihood of significant disease in a zonal fashion following international guidelines [21]. MpMRI of the prostate using this protocol has been shown to be able to rule out clinically significant disease with a negative predictive value at 90–95% in centres of excellence when compared to accurate reference tests [22], [23].

All men will need also to undergo accurate biopsy using the transperineal route. Both template prostate mapping with 5 mm sampling density and zonal template biopsy with targeted biopsy of MR-derived targets will be accepted. These tests have been shown to be comparable in terms of diagnostic accuracy and both yield a NPV again around 90–95% [24], [25], [26], [27].

While no single test can definitely rule out clinically significant disease, the combination of accurate imaging and histology minimises (although not eliminates) the possible miss-classification of untreated areas.

All men with a new diagnosis of prostate cancer are counseled about all standard radical therapeutic options. Those meeting the inclusion criteria for the NEAT trial are offered a patient information sheet, and to attend a screening and consent visit if interested. The consent, medical history, physical examination, and blood tests including a PSA test are collected. At baseline, a number of validated patient reported outcome measures (PROMs) are completed: International Prostate Symptom Score (IPSS) and IPSS Quality of Life (IPSS-QoL), 15-Item International Index of Erectile Function (IIEF-15), UCLA Expanded Prostate Cancer Index Composite (EPIC) urinary and bowel domains, EQ-5D Health related Quality-of-life, Functional Assessment of Cancer Therapy for Prostate (FACT-P) and Memorial Anxiety Scale for Prostate Cancer (MAX-PC) [28], [29], [30]. Patients are also asked whether they want to participate to an optional embedded qualitative study aiming to assess patients' satisfaction with alternative electronic forms of follow-up.

#### 2.3.2 Focal irreversible electroporation

**Fig. 3:**
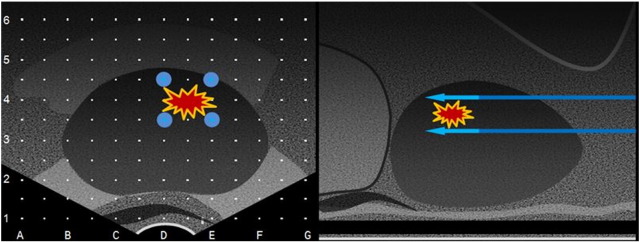
This a representative diagram of the treatment planning for irreversible electroporation of the prostate. Left and right images represent an axial and sagittal views of a TRUS, respectively. The electrodes are positioned around the lesion to treat (left image), highlighted as a red spot. A given active length exposure (blue arrow in the right image) is determined according to the longitudinal dimension of the lesion.

Under general anaesthesia and deep muscle paralysis, a suprapubic catheter or a urethral catheter, in case of contraindication to a suprapubic catheter, are inserted. The 19 G electrode-needles are inserted via a transperineal approach under transrectal ultrasound (TRUS) guidance using a brachytherapy grid. The electrical field in IRE is created by positioning electrodes to the margins of the tumour (Nanoknife™, AngioDynamics, New York, USA). The number of electrodes used is dependent on the size and the shape of the lesion to treat (Fig. 3). Usually, four electrodes are required to cover a discrete lesion within the prostate, although one additional electrode may be needed in larger lesions. The device will be set to deliver 90 pulses with a pulse length at 70 μs in order to achieve an electrical field between 20 and 40 A. This current has been shown to cause complete ablation in the target area without causing heating effect, which in turn is possible when the current is higher [31].

In this study, to allow precise placement of the electrode-needles, a MR/TRUS fusion device is used. Various studies have previously shown that integrating mpMRI images to real-time TRUS images can be beneficial to better target intra-prostatic tumours for biopsy and one previous study has shown that fusion is feasible with HIFU focal therapy [32], [33], [34]. In the NEAT trial we will use a high accuracy MR/TRUS fusion device (SmartTarget) that by non-rigid registration compensates for the distortion of the prostate [35], [36]. This fusion device is currently under development and commercialisation at UCL. Since this is a pilot study, and the accuracy of this software is yet to be defined for IRE focal therapy, the surgeon is free to accept or reject the treatment planning calculated by the software.

Once the electrodes have been positioned, the surgeon will measure the distances between the electrodes and will select the electrodes' active length according to the cranio-caudal length of the target area. Based on these two measurements which are manually entered in the device, the Nanoknife software calculates the voltage necessary to obtain the optimal electrical field (20–40 A). After the first 10 pulses have been delivered, the actual electrical field in the prostatic tissue is verified. If the electrical field is in the optimal range, then the remaining 80 pulses are delivered; otherwise, the voltage is modified as required.

The focal IRE treatment is planned as a day-case procedure. Men arrive in the morning of the procedure, and are usually discharged in the afternoon or in the evening. Catheter withdrawal is scheduled between 3 and 10 days at the same time of the early contrast MRI scan. Patients are prescribed pain killers, antibiotics and laxatives for one week after the procedure.

##### 2.3.2.1 Therapy escalation and rationale

Only men with MR-visible anterior disease will be treated. At this early stage of evaluation of this treatment, targeting the anterior part of the prostate is the safest approach since it is away from the rectum. As a consequence, rectal toxicity, and particularly the risk of recto-urethral fistula are minimised since no previous clinical study has accurately shown the extent of ablation that can be achieved with IRE.

In this prospective development study, we have incorporated a ‘dose-escalation or dose optimisation’ protocol based on target volume. Indeed, using a fixed algorithm including fixed pulse length and number of pulses, the delivered energy is mainly determined by the needle active length and by the needle distances, which in turn are both derived by the target volume. We are going to stratify the first nine patients into three groups of three patients each based on a maximum target volume. The ablation volume in millilitres has been calculated with respect to an average prostate volume, which corresponds to 40 ml. In the first group, a maximum of 4 ml ablation or target volume representing maximum 15% of the prostate volume is admitted. This represents the minimum amount of tissue that is estimated to be possible to ablate in focal therapy. The limit will be increased to 15 ml ablation or 40% prostate volume, and to 20 ml ablation or 50% prostate volume, for the second and third group, respectively. The upper threshold corresponds to the maximum amount of tissue it would be possible to treat in each subgroup of patients and not a target to achieve. In the remaining 11 patients, the upper threshold set at 20 ml ablation or 50% prostate volume will be respected.

#### 2.3.3 Follow-up

After discharge from the hospital, an early contrast-MRI is organised to assess the ablation of the target area, and to rule out significant damage to structures adjacent to the prostate. Outpatient visits will occur at 6 weeks, 3, 6, 9 and 12 months. At each, adverse events will be ascertained, PROMs used at baseline are filled and serum PSA level measured. At 6 months, all men undergo a mpMRI and targeted biopsy of the treated area. The biopsy density is set to at least 1 targeted biopsy per 1–2 ml residual prostate volume. Untreated areas will be sampled only if a new suspicious lesion is detected on the 6 months mpMRI or if there is a suspicious change in another area. This sampling strategy aims to minimise the possibility of biopsy-related adverse events by avoiding further sampling of untreated areas, which in the case of a negative mpMRI and a recent template negative biopsy is unlikely to harbour significant disease. As per standard practice, ‘for cause tests’ are permissible at any time point. For instance, a significant unexplained rise in the PSA may drive additional mpMRI scans and/or additional biopsy.

#### 2.3.4 Testing alternative forms of follow-up

Patients will be offered an optional embedded qualitative study. This aims to evaluate patient satisfaction in the use of telephone consultation as well as with electronic tools for filling questionnaires. We aim to collect pilot data on patients' satisfaction, usability and acceptability with the use of these tools offering different strategies at different time-points. Patients in this nested study will have a telephone consultation at 6 weeks and will receive their questionnaires at home by post. At 6 months, patients will have a normal clinic visit, but they will complete the questionnaires using a tablet hand-held computer in the research clinic prior to meeting the clinician. At 9 months, patients will have a telephone consultation and will complete online questionnaires. Finally, following the last clinic visit at 12 months, men in the embedded study will be interviewed using a standardized semi-structured questionnaire. The interviews will last around 30 min, and will be audiotaped and transcribed. Thematic extraction will be used to determine whether these men found the method of data collection acceptable, easy to use and would continue to use it. Direct anonymised quotes will be used to illustrate particular themes which evolve. The purpose of the embedded study will be to determine optimal and cost-effective follow-up methods in future studies.

#### 2.3.5 Stopping rules

In case a recto-urethral fistula, assessed clinically or radiologically, occurs in any one patient at any time, the IDMC will review the case and make recommendations to the TSC. Any further treatments will be halted while this occurs. We envisage that the target volume will be reduced to the preceding maximum volume. If a recto-urethral fistula occurs in more than one patient, the IDMC and TSC will evaluate whether it is safe to proceed with the trial.

#### 2.3.6 Objectives

The primary objective is to determine the adverse events, genito-urinary and rectal side-effect profile at 12 months after focal IRE.

There are a number of secondary objectives included in the study. First, to collect pilot data on the disease control rates of focal IRE for the treatment of prostate cancer localised in the anterior part of the prostate using post-treatment biopsy. Second, to report the rate of patients achieving a trifecta (pad-free/leak-free urinary continence, erections sufficient for penetrative intercourse, and absence of clinically significant disease in the treatment area). Third, to determine domain-specific genito-urinary, rectal toxicity and health-related quality of life after IRE. Fourth, to obtain pilot data on the utility of mpMRI in the follow-up of focal IRE. Fifth, to explore the utility of using a MR/TRUS fusion device for delivering focal IRE. Finally, in men participating within the embedded qualitative study, we will assess patient usability, acceptability and satisfaction in the use of electronic tools and in telephone consultations.

#### 2.3.7 Statistical analysis

##### 2.3.7.1 Sample size calculation

As the primary objective of the study is to determine the toxicity profile of focal IRE, the sample size was calculated on the basis of common adverse events, which are urinary incontinence and erectile dysfunction. Further, since the results could be used to progress to a IDEAL stage 2b multi-centre therapeutic confirmatory study with 2–3 years of follow-up, it is essential to have a sample size that gives appropriate precision around these key outcomes. Considering an expected proportion of pad-free/leak-free urinary continence of 95%, and a rate at 10% of erectile dysfunction, the ±95% CI around such proportions with n = 10, n = 20 and n = 25 would be at 13.5%, 9.55% and 8.5% for incontinence, and at for 18.6%, 13.2% and 11.8% for erectile dysfunction, respectively. We have therefore chosen to set the sample size at n = 20, as there is significant increased precision from n = 10 (Δ = 3.95% and 5.4%), but little improvement is achieved if more patients are included (Δ = 1.05 and 1.4%).

##### 2.3.7.2 Primary outcome

Primary outcome will be measured by PROMs evaluating sexual, urinary and rectal toxicity. Sexual outcome will be estimated by the rate of men with erectile dysfunction, defined by an inability to have erections sufficient for penetrative intercourse, as measured by the IIEF-15 questionnaire. Primary and secondary outcomes will be reported along with 95% confidence intervals.

##### 2.3.7.3 Secondary outcomes

Various outcome measures will be used according to the specific outcome of interest. The rate of clinically significant disease and of any disease will be calculated using the findings of targeted biopsy of the treated area at 6 months. Adding this rate to sexual and continence primary outcomes, we will estimate the rate of trifecta status achievement. The proportion of domain-specific genito-urinary and rectal toxicity as well as health-related quality of life will be estimated using validated questionnaires (IPSS, IPSS-QoL, IIEF-15, UCLA-EPIC urinary and bowel domain, EQ-5D QoL, FACT-P and MAX-PC). The utility of mpMRI in detecting residual/recurrent disease will be evaluated against follow-up biopsy (reference test) to calculate point estimates of sensitivity, specificity, negative and positive predictive values. Pilot data on the utility of MR/TRUS fusion device for delivering focal IRE will be evaluated by the proportion of men in which the planned treatment volume was changed. The use of electronic tools and of telephone follow-up after treatment will be evaluated by a qualitative thematic analysis.

## 3 Discussion

NEAT is an IDEAL prospective development 2a study. As opposed to new drug development, in which the pathway is rigorous, established and protected by strict observance of rules, the assessment of new technologies in surgery is recognised to be more fluid, less rigorous, and commonly based on retrospective rather than on prospective study designs. There are a number of factors that make surgery a more difficult field in which to apply multi-step rigorous study designs: the complexity of interventions, the learning curve, and the surgeons' and patients' equipoise. The recognition of these issues has led to the development of a specific pathway for the evaluation of novel surgical procedures, which is more feasible in the surgical environment, and at the same time ensures patients' safety and meaningful results [13], [37], [38].

Following on from pre-clinical studies testing IRE and one stage I trial, NEAT represents the next natural step in the assessment of this new technology for treating localised prostate cancer. The study population, the intervention and the selected outcomes have been chosen to achieve meaningful results by which to determine whether a larger trial is necessary.

One area that requires further elaboration is the definition of what constitutes clinically significant prostate cancer. In other words, disease that would otherwise impact on a man's quality of life or life expectancy, if left untreated. There is no general consensus on what represents clinically significant prostate cancer, but it is well recognised that most prostate cancers do not impact on survival. Nonetheless, there is strong evidence that tumour stage, grade and volume are substantial features of clinically significant disease. In localised prostate cancer, Gleason grade is the most significant predictor of mortality. In large cohort studies, patients with pure Gleason 3 + 3 had rarely metastasised or died from their disease over a 15 year period of follow-up [4], [39]. As in other malignancies, cancer volume is also a determinant of aggressiveness, but the threshold volume has not been determined. Historically, thresholds between 0.2 and 0.5 ml have been considered significant, although one recent study based on the findings of a large European RCT (ERSPC) has shown that this volume may be up to 1.3 ml in a screened population [40], [41]. We will carefully select men using state-of-the-art diagnostic tests (mpMRI and template with targeted biopsy) that have the ability to minimise the possibility of disease misclassification and leaving clinically significant disease untreated. The strict histological classification used for significant disease in particular predicts the presence of a lesion with volume ≥ 0.2 ml with over 95% sensitivity [42]. The population of men is therefore likely to benefit most from undergoing a tissue-preserving approach that can potentially combine the oncological benefit with preservation of function. In addition, the selection of only men with anterior disease makes the possibility of recto-urethral fistula, which represents the most significant complication after focal therapy in prostate cancer, very unlikely to happen.

IRE is a procedure with potentially attractive characteristics which may be ideal for delivering focal therapy in the prostate especially as it has been shown to have tissue-selectivity in pre-clinical models [8]. Animal studies have demonstrated that the application of therapeutic IRE led to homogeneous ablation of prostatic tissue with preservation of collagenous structures, which in the prostate means the urethral sphincter, the neurovascular bundles, and the urethra [11]. If this was true in clinical studies such as NEAT, the most significant side-effects of prostate treatments, namely urinary incontinence and erectile dysfunction may be further minimised. The application of IRE in a homogeneous group of men in a standardized fashion will assist to appropriately verify these aspects of the technology.

Some discussions of the secondary efficacy parameters that we propose to use are warranted here. Most previous studies assessing the efficacy of various sources of energy to deliver focal therapy in the prostate have been negatively impacted by the absence of systematic assessment of ablative success or failure [6]. This is a key methodological problem in the assessment of treatments for prostate cancer. Indeed, as the natural history is prolonged, and these early studies have short to medium follow-up, survival is difficult to include as an outcome measure of oncological efficacy, so the incorporation of reliable surrogate outcome measures has gained some merit [6]. While PSA is considered a valid outcome measure for radical prostate cancer treatments, it is not validated after focal therapy and due to the ongoing secretion of PSA by untreated tissue might not be valuable. Also, although the selection of only men with MR-visible lesions makes the detection of oncological failure potentially easier with imaging, mpMRI is also not validated as a measure of local control after IRE although studies have shown its utility after HIFU and cryotherapy [43], [44]. As a consequence of these comments, the only reliable measure of ablation remains the histological analysis of the tissue in the treatment area, which in this study in included with a high sampling density.

### 3.1 Study limitations

The first limitation is linked to one of the advantages for patients in terms of safety, which is the anterior location of the prostatic cancer as this limits external validity of the treatment since around two-thirds of prostatic cancers are located in the posterior prostate which is histologically different to the anterior prostate and thus IRE may have different effect. Thus, as the successful treatment of the target area is closely linked to the inner electrical characteristics of that target, the success or the failure of the treatment of the anterior prostate would not extrapolate to a similar result if the same treatment were applied to the posterior prostate.

Further, the study is a single centre trial in a tertiary hospital particularly expert with focal therapy and needle-based transperineal prostate procedures; therefore, the results of this study are unlikely to allow wide generalization. While we acknowledge this limitation, and clearly multi-centre studies will be necessary, the single centre expert setting is the preferred context in which to allow ideal development of new technologies.

Another limitation may be the validity of PROMs filled using alternative methods, for those men participating to the nested qualitative study. Validated PROMs represent robust instruments for assessing a given outcome, but paper-based questionnaires completed during a clinic visit may not represent the optimal manner by which to collect them. Also, clinic visits themselves, although necessary in a trial setting, when part of standard care if a trial determined this so, oblige patients to physically come every time, which is both time-consuming and expensive for the patient and expensive for a healthcare system. The use of telephone follow-up and of electronic tools might address this timely issue and various models have been shown to be successful [45]. However, the validity of such tools has not been compared to traditional paper-based questionnaires in the setting of experimental prostate cancer treatments. There might be a verification bias as men might respond differently when using such tools. While we accept this as a limitation, we believe this study is justified by the need for determining the satisfaction of men with these tools, which if positive can enhance trials feasibility. In addition, all the electronic tools were used in interim visits, whereas baseline and last-visit PROMs were completed in a traditional manner. Thus, the variation in time might be affected, but the final outcome will not.

## 4 Conclusion

The NEAT trial will allow an appropriate evaluation of focal IRE in men with localised prostate cancer by using validated patient reported outcomes and systematic assessment of local efficacy. The outcomes of NEAT may be used to progress to a large confirmatory multi-centre trial. This adaptive design may provide guidance in the challenging landscape of health technology assessment in prostate cancer therapy.

## Conflict of interest

M. Valerio has received funding for conference attendance from Geoscan Medical and from AngioDynamics. M. Emberton and H.U. Ahmed receive funding from Sonacare, GSK and Advanced Medical Diagnostics for clinical trials. M. Emberton is a paid consultant to Steba Biotech, AngioDynamics and SonaCare Medical (previously called USHIFU). H. U. Ahmed is a paid consultant to Sonacare Medical (through membership of Data Monitoring and Safety Board for a clinical trial using the Sonablate™500 HIFU device in the USA). Both have previously received consultancy payments from Oncura/GE Healthcare and Steba Biotech. L. Dickinson has received trial funding support from SonaCare Medical and previously consultancy fees from SonaCare Medical and Oncura. None of these sources had any input whatsoever into this article.
